# Preserving the posterior cortex of the sternum during resection of a superficial anterior chest wall sarcoma

**DOI:** 10.1093/jscr/rjab450

**Published:** 2021-10-28

**Authors:** Akio Sakamoto, Itaru Tsuge, Takashi Noguchi, Shuichi Matsuda

**Affiliations:** The Department of Orthopaedic Surgery, Graduate School of Medicine, Kyoto University, Kyoto, Japan; Department of Plastic and Reconstructive Surgery, Graduate School of Medicine, Kyoto University, Kyoto, Japan; The Department of Orthopaedic Surgery, Graduate School of Medicine, Kyoto University, Kyoto, Japan; The Department of Orthopaedic Surgery, Graduate School of Medicine, Kyoto University, Kyoto, Japan

## Abstract

Following resection of a sternal tumor, respiratory dysfunction can occur and rigid reconstruction is necessary. An 82-year-old woman noted a mass in the anterior chest wall that was increasing in size. The tumor was located on the left aspect of the sternum at the level of the third rib. A radiation-induced malignant spindle cell tumor was diagnosed because of a history of irradiation for hilar lymph node carcinoma. The tumor was resected with the surrounding tissues of the second-to-fourth ribs and sternum. The posterior sternal cortex was preserved by cutting with a curved chisel under fluoroscopy. The chest wall defect was reconstructed with a 2-mm thick Gore-Tex® sheet and a local transpositional flap. Sternal resection with a chisel under fluoroscopy avoids damage to the internal thoracic artery. Preserving the posterior sternal cortex does not require rigid reconstruction. The procedure is minimally invasive.

## INTRODUCTION

Sternal tumor resection following an anterior chest wall defect results in severe respiratory and circulatory compromise [1]. To avoid respiratory dysfunction, rigid reconstruction is necessary after resection of a sternum tumor [1]. Indeed, titanium plates have been widely used for this purpose [2, 3] because titanium plates are biocompatible [4] and can be shaped to fit thoracic defects [5].

In the current report, the posterior sternal cortex was preserved after a chest wall sarcoma resection without the need for rigid reconstruction. The case was complicated by a postoperative wound infection. Non-rigid reconstruction is a minimally invasive procedure and facilitates the treatment of wound infections.

## CASE PRESENTATION

An 82-year-old woman sought evaluation of a mass in the anterior chest wall. A cardiac pacemaker was placed for bradycardia due to atrial fibrillation 3 years before. Twenty years prior an enlarged hilar lymph node was biopsied and the histologic evaluation revealed an adenocarcinoma that was thought to be a metastatic lung carcinoma, but the origin was not established.

**Figure 1:**
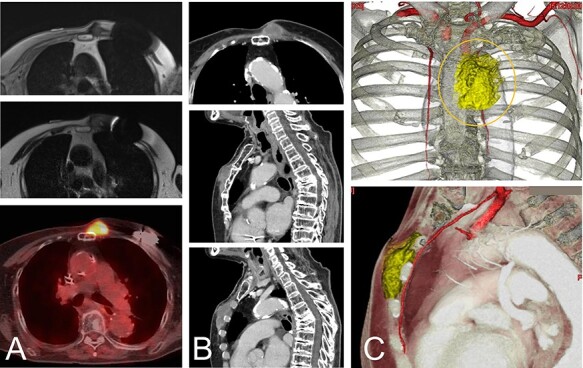
An 82-year-old woman with a malignant spindle cell sarcoma in the superficial chest wall. The MRI shows a lesion involving the anterior sternum on the left side with low-to-intermediate signal intensity on T1- (**A**-top) and T2-weighted images (A-middle) with a high SUVmax on FDG-PET (A-bottom). CT with contrast medium depicted the lesion (**B**). The lesion involved not only the anterior sternum (B-top and -middle), but also the intercostal space (B-bottom). The cut line (orange line) is drawn with a yellow line. The internal thoracic artery runs under the tumor and the vessels were resected together (**C**).

A magnetic resonance image (MRI) and computed tomography (CT) showed the chest wall lesion on the surface of the sternum and the distal third rib. The tumor size was 4 x 5 cm, and the lesion extended to the subcutaneous tissue and intracoastal spaces between the second and third ribs (Fig. 1). The histologic evaluation of a biopsy specimen was characterized as proliferation of atypical spindle cells.

The tumor was resected along with the surrounding normal tissues of the overlying skin, the surrounding subcutaneous tissues, and the pectoralis major muscle. The anterior cortex of the sternum at the proximal and distal cut lines, as well as right lateral aspect of the cortex was cut with a high-speed burr. The left second-to-fourth ribs were cut along the resection line and an additional excision of ~5 mm was made at each second-to-fourth rib to confirm complete pleural reaction and handle the lesion for the next step.

Under fluoroscopy the left outline of the sternum was confirmed. As a tip, a 1.5-mm Kirschner wire was placed along the line of the sternum on the skin. A curved pelvis osteotomy chisel was used to cut the sternum from right-to-left, then the blade was used along the anterior surface of the posterior cortex of the sternum. Under fluoroscopy guidance, the blade was directed over the left outline of the sternum, which was marked with a Kirschner wire. Incomplete resection of the left outline of the sternum was cut with the curved chisel from the distal end. The remaining soft tissues, including the internal thoracic arteries and veins, were cut and ligated while the lesion was resected.

**Figure 2:**
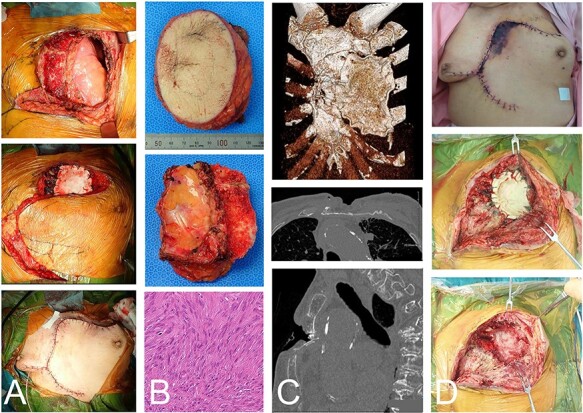
The same case as in Figure 1 with a malignant spindle cell sarcoma in the superficial chest wall. The tumor and partial sternum resection was performed with the second-to-fourth ribs, leaving the posterior cortex of the sternum (**A**-top). Gore-Tex® covers the chest wall defect and the local transposition flap was elevated (A-middle and -bottom). The resected material shows the chest wall and the bisected sternum (**B**-top and -middle). The histologic evaluation shows spindle cell proliferation arranged in fascicles with a storiform pattern and atypia (B-bottom). CT depicted the remaining anterior bones and Gore-Tex® sheet (**C**-top). The posterior cortex was preserved (C-middle). A fracture, possibly due to spinal kyphosis, occurred 3 months after the operation (C-bottom). The edge of the flap was necrotic (**D**-top). The wound was opened (D-middle) and the Gore-Tex® sheet was removed. Fibrous reactive tissue was confirmed on the surface of the lung (D-bottom).

The skin defect was 10 x 8 cm, but the dorsal sternal cortex was preserved and the chest wall defect was 10 x 5 cm on the left chest wall. Consequently, a 2 mm-thick Gore-Tex® Soft Tissue Patch (W. L. Gore & Associates, Newark, DE, USA), a pure and unique expanded polytetrafluoroethylene prosthesis, was placed and sutured to the chest wall. A local transpositional flap was elevated for soft tissue reconstruction. No paradoxical respiration was observed postoperatively. The pathologic diagnosis on the resected material was a spindle cell sarcoma. Considering the clinical history of irradiation, a radiation-induced sarcoma was diagnosed (Fig. 2).

The edge of the flap was necrotic with a *Pseudomonas aeruginosa* infection. To treat the infection, the Gore-Tex® sheet was removed. Under the Gore-Tex® fibrous reactive tissue was noted on the surface of the lung (Fig. 2). The lung did not collapse after removal of the Gore-Tex®, thus additional reconstruction was not performed. The wound was left open and the infection was treated with antibiotics. After the infection had resolved, the wound was closed with suture.

## DISCUSSION

In resecting a malignant tumor on the superficial chest wall, the anterior cortex of the sternum provides a sufficient margin for complete resection. In our case, the sternum was easily cut with a curved chisel under fluoroscopy. Without preserving the sternal posterior cortex, rigid reconstruction would be required because full thickness anterior chest wall defects after sternal tumor resection require reconstruction to obtain structural stability and avoid paradoxical respiration [6].

Gore-Tex® prosthesis coverage is used with titanium plates for anterior chest wall defects [2, 3]. A Gore-Tex® Soft Tissue Patch was previously reported to be strongly adherent to and incorporated in the adjacent host tissue [7]. Fibrous reactive tissue was confirmed on the surface of the lung after removal of Gore-Tex® in this case. The fibrous tissue played a role in covering the chest wall defect and in preventing infection to the thoracic cavity. It is possible that the Gore-Tex® incited the formation of fibrous tissue, which is a common phenomenon.

In the current case, the rotation flap was a random pattern flap, partially failed and was necrotic. Prosthetic materials, including the Gore-Tex® prosthesis, carry the risk of subsequent infection because the materials are not incorporated into the host tissue [6, 8]. To treat the infection, the necrotic tissue, as well as the infected Gore-Tex®, were removed. In the current case, a random pattern flap appeared not to be ideal under tension, especially with the history of radiation, which was not ideal for reconstructing the chest wall defect. A vascular flap was reliable in the current case. Furthermore, to seal off the lung, an autologous repair with fascia or dermis might have been safer than mesh and coverage with muscle-facial flaps. Of note, the non-rigid reconstruction had a positive role in the treatment of infection. Moreover, the posterior cortex of the sternum was thought to prevent infection from spreading to the mediastinum.

In the current case report, the posterior cortex of the sternum was preserved during resection of an anterior chest wall tumor. The posterior cortex prevents infection from spreading to mediastinum. Posterior sternal cortical preservation is a minimally invasive procedure. Non-rigid reconstruction has merits in treating infections in such cases.

## CONFLICT OF INTEREST STATEMENT

None declared.

## FUNDING

None.
